# Abdominal Massage Improves the Symptoms of Irritable Bowel Syndrome by Regulating Mast Cells via the Trypase-PAR2-PKC*ε* Pathway in Rats

**DOI:** 10.1155/2022/8331439

**Published:** 2022-09-28

**Authors:** Huanan Li, Wei Zhang, Fei Ma, Xiaofan Zhang, Yuyan Wang, Jingui Wang

**Affiliations:** ^1^Department of Tuina, First Teaching Hospital of Tianjin University of Traditional Chinese Medicine, National Clinical Research Center for Chinese Medicine Acupuncture and Moxibustion, Tianjin 300000, China; ^2^Tianjin Academy of Traditional Chinese Medicine Affiliated Hospital, Tianjin 300120, China

## Abstract

**Background:**

Irritable bowel syndrome (IBS) is a clinical disease mainly characterized as a syndrome of abdominal pain and discomfort, which frequently occurs in humans aged 20–50. Abdomen massage is of great medical significance for the health of the human body, including promoting intestinal peristalsis, relieving constipation, and facilitating weight loss. However, its potential benefits in alleviating IBS and the underlying mechanisms remain elusive.

**Methods:**

In this study, we established an IBS model in rats to evaluate the effects of abdomen massage. Forty male Sprague Dawley (SD) rats were randomly assigned into 4 groups: the normal (control) group, IBS group, abdominal massage group, and abdominal massage + ketotifen treatment group (*n* = 10 rats in each group). Abdominal massage was performed once a day for 5 minutes for 14 days. On day 14, the rats were euthanized and the tissues were analyzed by transmission electron microscopy (TEM), immunohistochemistry or immunofluorescence staining, and laser confocal focus to visualize the micromorphology of the intestinal mucosa. The expression of TRPV1 and the release of trypase were determined by RT-qPCR and western blot.

**Results:**

We found that compared with the control group, the mast cells in the IBS group were significantly increased and the increased MC was partially decreased by an abdominal massage with or without ketotifen treatment. We also found that TRPV1 was upregulated in the IBS group. Abdominal massage with or without ketotifen treatment could attenuate the upregulation of TRPV1 in IBS. Mechanically, results of IHC and western Blot suggested that abdominal massage reduces the sensitivity of IBS by regulating the trypase-PAR2-PKC*ε* pathway.

**Conclusion:**

Overall, our results suggested that abdominal massage produces a beneficial effect in improving the symptoms of IBS through reducing mast cell recruitment and attenuating the trypase-PAR2-PKC*ε* pathway. Ketotifen could promote the effect of abdominal massage on IBS treatment, which can serve as a potential therapeutic strategy for IBS.

## 1. Introduction

Irritable bowel syndrome (IBS) is a group of intestinal dysfunction disorders characterized by the persistent or intermittent onset of abdominal pain, abdominal distension, and bowel habit changes in the absence of gastrointestinal tract structure and biochemical abnormalities. The incidence of IBS in Western countries is estimated to be 10%–20% [1]. Although the etiology and pathogenesis of IBS are not very clear, the role of mast cell-mediated mucosal changes has been implicated in the onset of IBS and has received extensive attention [2, 3]. However, whether massage can regulate the infiltration of mast cells into bowel tissues needs to be clarified.

Massage is an integral part of traditional Chinese medicine, which has been widely employed to treat chronic diseases, mitigate fatigue, and enhance physical fitness [4]. As an effective strategy for health care, massage is conducive to the relaxation of the tendon, activation of collateral ligaments, blood circulation enhancement, and pain relief [5]. Chinese medicine massage manipulation on the body surface and the local meridians can also generate a beneficial effect on the viscera including the abdomen. However, its potential benefits in alleviating IBS and the underlying mechanisms remain to be investigated.

PAR2, a G protein-coupled receptor composed of 397 amino acids, is widely expressed in gastrointestinal nerves and involved in pathophysiological reactions such as gastrointestinal motional changes, intestinal endocrine, and visceral pain [6]. It was reported that PAR2 could be activated by trypsin-like enzymes secreted by mast cells. PKC can phosphorylate various substrates which are reported to mediate cell division, proliferation, apoptosis, and cytoskeleton proteins [7]. PKC is widely expressed in peripheral sensory nerves, and PKC*ε* plays an important role in inflammation-induced hyperalgesia through the phosphorylation of TRPV1 [8]. Whether traditional Chinese medicine massage could regulate the PAR2/PKC pathway in IBS needs to be explored.

In this exploratory study, we aim to evaluate the abdominal massage and ketotifen treatment in a rat IBS model. We determined the quantity of mast cell infiltration by histological analysis, examined the microstructural of the colonic mucosa, and determined TRPV1 expression in the guts of each group. We demonstrated that abdominal massage improved the symptoms of IBS by reducing mast cell recruitment and downregulating the trypase-PAR2-PKC*ε* pathway. Ketotifen could enhance the beneficial effect of abdominal massage on IBS treatment. Our data not only propose massage and ketotifen treatment as a potential therapeutic strategy for IBS but also suggest that the activation of the trypase-PAR2-PKC*ε* pathway is associated with the onset of IBS syndrome.

## 2. Methods

### 2.1. Animal Models

Forty male Sprague Dawley (SD) rats with a body weight of 180–220 g (8 weeks old) were bought from Sipford Biotechnology Co., Ltd., and raised in an SPF environment of light cycle 12 h/12 h (light on time) and relative humidity 60%. All the rats were free for drinking water and a normal diet for 3 days and then randomly divided into 4 groups based on body weight: the normal (control) group, IBS group, abdominal massage group, and abdominal massage + ketotifen treatment group (*n* = 10 rats in each group). The randomization was based on a simple biased coin method. Each group of rats was allocated to a separate cage, and each rat is considered an experimental unit in each group. The blinding method was employed for all the people handling the mice until the end of the experiments. Hypothesis testing: two-sample inference estimation of sample size and power for comparing two means was employed to determine the sample size based on the mean of the population, sigma (common standard deviation), *α* (type I error rate) = 0.05, and the power = 0.80 (https://www.stat.ubc.ca/∼rollin/stats/ssize/n2.html). The IBS rat model was established as previously described [9]. The fasted rats were anesthetized, and enema was administered with acetic acid (4%, 1 mL/rat). The colon was then irrigated with 1 mL phosphate-buffered saline (PBS) to dilute acetic acid. The normal group was given 1 mL PBS. Abdominal massage was performed in “Guan Yuan, Zhongwan acupuncture clinics” for 5 minutes per day for a period of 14 days. The acupressure point was at the abdomen of the rats, and the massage was performed by an experienced practitioner. Animals that developed severe symptoms or more than 30% weight loss during the experiment were excluded from the analysis. In our experiment, all the animals were included for final analysis. Animals were euthanized by carbon dioxide asphyxiation on day 14 for subsequent analysis. Animal experiments were conducted in accordance with the guidelines for the care and use of laboratory animals developed by the National Institutes of Health. All the animal experiments gained the approval of the animal use and care committee of the First Teaching Hospital of Tianjin University of Traditional Chinese Medicine (20210312).

### 2.2. Histological Assessment and Mast Cell Counts

Tissue samples were fixed in 4% paraformaldehyde, dehydrated, and embedded in paraffin. Continuous sections were made with a thickness of 5 *μ*m and a distance of 30 *μ*m. Toluidine blue staining was performed after routine dewaxing, and images were captured under a Leica AM6000 microscope. The number of MCs was counted with Image J software.

### 2.3. RT-qPCR

Tissue RNA was extracted with Trizol reagent and then reverse transcribed into cDNA. Quantitative real-time PCR was performed with a SYBR Premix Ex Taq kit (TaKaRa) in a 7500 real-time PCR System (Applied Biosystems/Life Technologies, Carlsbad, CA, USA). The PCR cycling condition used 98°C 5 min, 40 cycles of 98°C 30 sec, 60°C 45 sec, and 72°C 60 sec, with signal detection at the end of each cycle. Finally, the 2^–∆∆Ct^ method was used to analyze the relative expression level and GAPDH was used as the internal reference gene. Primers used in this study were as follows: GAPDH-F: CGAGATCCCTCCAAAATCAA; GAPDH-R: TTCACACCCATGACGAACAT; TRPV1-F: CGCGGCGTGGGGAAAGACAT; TRPV1-R: CCTTCGGCTGCGGCGTGAT.

### 2.4. Western Blot

Total protein was extracted from tissues using RIPA lysis buffer, and the supernatant containing total protein lysate was quantified by using a BCA Protein assay kit. An equal amount of protein samples were added into polyacrylamide gel for electrophoresis, followed by semidry membrane transfer. 5% skimmed milk was used to block nonspecific binding sites on the membrane before the incubation of anti-TRPV1 antibody (1 : 500), anti-PAR2 antibody (1 : 400), anti-PKC*ε* antibody (1 : 1000), anti-PAR2 antibody (1 : 500), and anti-PIP2 antibody (1 : 400) (Abcam, USA) overnight at 4°C. The membrane was further labeled with HRP secondary antibody, and protein bands were developed using a DAB chromogenic kit (Abcam, USA). The grey value of the protein band was analyzed with ImageJ software and normalized to GAPDH or *β*-actin.

### 2.5. Immunohistochemistry Staining

Paraffin-embedded tissues were sectioned continuously, and the tissue sections were dehydrated with gradient alcohol solution and sealed with 3% H_2_O_2_. After blocking with normal goat serum, the sections were stained with primary antibody of tryptase (Abcam, USA) and HRP-second antibody. Diaminobenzidine solution was added to the sections for color development, and the expression level of tryptase was observed under a microscope.

### 2.6. Immunofluorescence Staining and Confocal Laser Scanning

The rats were anesthetized, and 10∼15 points at a colon-bladder level were exposed at about 6 cm away. Fluorescent dye 1, 1′-bis (octanoyl)-3, 3, 3′, 3′-tetramethylindole carbonyl anthocyanin perchlorate (DiI, InvitrogenTM, DiI) was injected into each point (Thermo Fisher Scientific Inc.). In order to prevent dye leakage and the contamination of adjacent organs, the injection needle was kept for 1 min, and each point was cleaned with 0.9% NaCl solution after injection. One week after the DiI injection, the small intestine tissues were quickly separated, fixed in 4% paraformaldehyde solution for 6–8 h, and precipitated in 30% sucrose solution at 4°C. The sections were blocked with 10% rabbit serum for 1 h, and stained with sheep anti-rat TRPV1 polyclonal antibody (1 : 100 dilution) was added at 4°C overnight, followed by the staining of FITC-labeled rabbit anti-sheep IgG/rabbit anti-Rat IgG (H + L) Secondary Antibody, Texas Red (1 : 50 dilution) at 37°C for 30 min. The section was counterstained with DAPI and the sections were sealed with glycerin. A laser confocal microscope was used to quantify the percentage of TRPV1-positive cells in total DiI labeled cells by randomly taking 3 sections from each paraffin block and 3 fields from each section.

### 2.7. Transmission Electron Microscopy (TEM)

Briefly, the tissue mass was fixed with 2.5% glutaraldehyde in phosphate buffer preparation for 2 hours and then rinsed with 0.1 M phosphoric acid rinse solution. The tissue mass was then dehydrated in an alcohol gradient and embedded. After the section, the slice was stained with citrate before electron microscope observation.

### 2.8. Statistical Analyses

Data were presented in means ± standard deviation. GraphPad Prism 8 software was used for statistical analyses and photography. Samples were subjected to the Kolmogorov–Smirnov normality test. Only the IBS group passed the normality test. The primary results are the mast cell filtration score. All the other parameters are secondary outcomes. The comparison among multiple groups was performed by one-way ANOVA, with Tukey's test as the post hoc test. *P* < 0.05 was considered to be statistically significant.

## 3. Results

### 3.1. The Role of Mast Cells (MCs) in the Treatment of Visceral Hypersensitivity in IBS by Abdominal Massage

**Figure 1:**
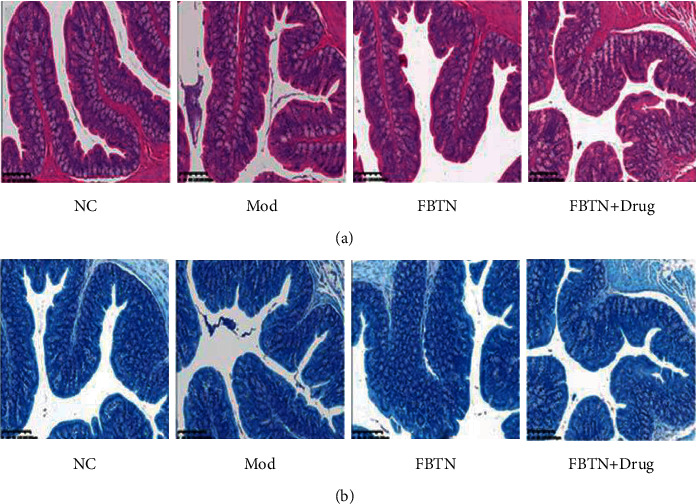
Abdominal massage attenuated the intestinal damages and infiltration of MC. (a) Morphological changes of small intestinal mucosa were detected by HE in normal (NC), IBS (mod), abdominal massage (FBTN), and ketotifen treatment groups (FBTN + drug) (scale bar 50 *μ*m). (b) Mast cells were detected by toluidine blue staining in normal, IBS, abdominal massage, and ketotifen treatment groups (scale bar 50 *μ*m).

The IBS rat model was successfully established, as evidenced by the results of HE staining that the morphology of intestine tissues in the IBS group showed different degrees of damage when compared to that in normal rats (Figure 1(a)). IBS tissues displayed loosely arranged villi with inflammatory cell infiltration. The administration of abdominal massage (FBTN group) restored the normal intestine tissue morphology, as evidenced by the long and complete villi, and the orderly arrangement of intestinal epithelial cells. When IBS rats were treated with massage and mast cell blocker ketotifen (FBTN + Drug group), the protective effect of abdominal massage seemed to be abolished, as reflected by the swollen mucous membrane and acinar cells (Figure 1(a)). As shown in the picture of toluidine blue staining, compared with the normal control group, the quantity of MC in the IBS group was significantly increased, which can be reduced after the administration of abdominal massage. When IBS rats were treated with abdominal massage and ketotifen together, the counts of MC were further reduced (Figure 1(b)). Together, these results suggest abdominal massage improves IBS by reducing MC infiltration in the intestine tissues.

### 3.2. Abdominal Massage Protected the Microstructure of the Colonic Mucosa

**Figure 2:**
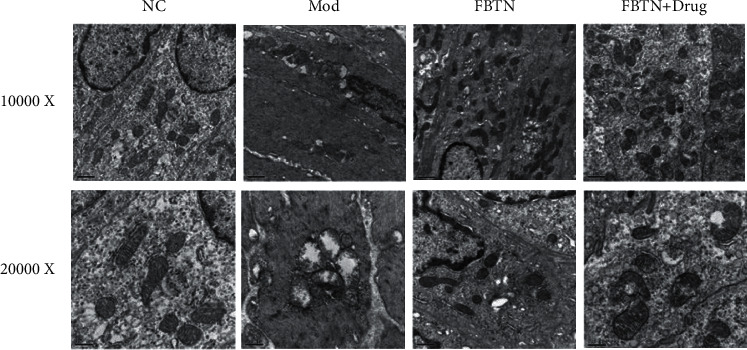
Abdominal massage protected the integrity of mitochondria in cells of the colonic mucosa. (a) Evaluation of cellular microstructures in the colonic mucosa by transmission electron microscopy (TEM, upper panel: 10000*x*, scale bar 0.50 *μ*m, down panel: 20000*x*, scale bar 0.25 *μ*m).

It is well known that mitochondria are not only involved in cellular energy metabolism but also mediate the cellular inflammatory response. Subsequently, transmission electron microscopy (TEM) was used to examine the effect of IBS and massage on intestinal mucosal mitochondria. As shown in Figure 2, compared with the control group, the number of mitochondria in the IBS group was significantly reduced, and mitochondria in the IBS group showed a swollen morphology, with the internal cristae being disrupted. The administration of abdominal massage in the FBTN group protected mitochondrial integrity in the intestinal mucosa of the IBS rat model. Furthermore, this effect of massage was enhanced when MCs were blocked with ketotifen (Figure 2).

### 3.3. Effect of Abdominal Massage on TRPV1 Expression

**Figure 3:**
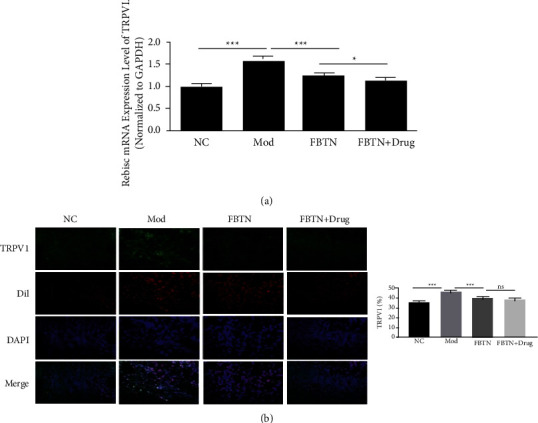
Effect of abdominal massage on TRPV1 expression. (a) Relative mRNA expression of TRPV1 in small intestinal mucosa tissues from the normal, IBS, abdominal massage, and ketotifen treatment groups. (b) Immunofluorescence (IF) images of TRPV1 staining in small intestinal mucosa tissue from the normal, IBS, abdominal massage, and ketotifen treatment groups (left panel) and percentage of TRPV1-positive staining (right panel). ^*∗*^*P* < 0.05, ^*∗∗*^*P* < 0.01, ^*∗∗∗*^*P* < 0.001.

Transient receptor potential cation channel subfamily V member 1 (TRPV1) was reported to be a noxious receptor and a signaling transducer to affect mitochondrial function. Inhibition of TRPV1 plays an important role in improving gastrointestinal function and reducing visceral hypersensitivity in rats [10, 11]. We next detected the expression of TRPV1 with RT-qPCR and found that TRPV1 was upregulated in the IBS group compared to the control group (*p*=0.1269, Figure 3(a)). Abdominal massage could attenuate the upregulation of TRPV1 in the massage group when compared to the IBS group (*p* < 0.001, Figure 3(a)). The application of an MC blocker could further inhibit the expression of TRPV1 (*p*=0.0205, Figure 3(a)). The protein level of TRPV1 was examined with immunofluorescence. As shown in Figure 3(b), TRPV1 located in the plasma membrane of the cells was highly expressed in the IBS group (*p* < 0.001, Figure 3(b)), and TRPV1 expression was suppressed by abdominal massage (*p* < 0.001, Figure 3(b)). The presence of MC blocker seemed to further reduce trpv1 protein level, although the difference was not significant (*p*=0.1329, Figure 3(b)). The abovementioned results indicate that abdominal massage could suppress IBS-induced TRPV1 expression.

### 3.4. Abdominal Massage Regulates the Trypase-PAR2-PKC*ε* Axis

**Figure 4:**
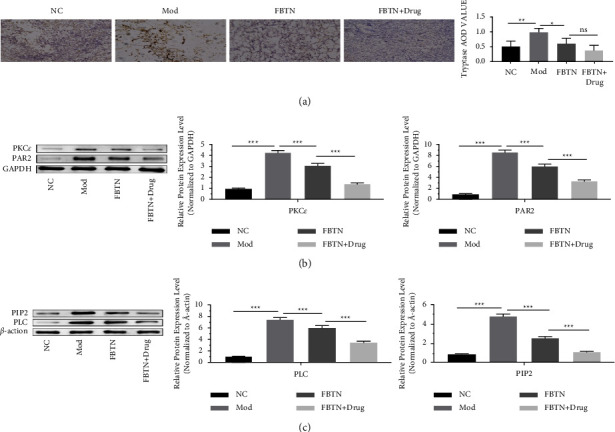
Abdominal massage attenuated IBS-induced activation of the trypase-PAR2-PKC*ε* pathway. (a) Representative images of trypase IHC staining (left panel) and trypase AOD value analyzed with ImageJ software (right panel). (b) Detection of PAR2 and PKC protein levels in small intestinal mucosa tissues from the normal, IBS, abdominal massage, and ketotifen treatment groups by western blot (left panel: representative images of PAR2 and PKC western blot; middle panel, a summary of relative PKC levels in four groups; right panel, a summary of relative protein levels of PAR2 in four groups. Data were normalized to GAPDH). (c) Detection of PLC and PIP2 protein levels in small intestinal mucosa tissues from the normal, IBS, abdominal massage, and ketotifen treatment groups by western blot (right panel: representative blots of PLC and PIP2; middle panel, a summary of relative PLC levels in four groups; right panel, a summary of relative protein levels of PIP2 in four groups. Data were normalized to GAPDH). ^*∗*^*P* < 0.05, ^*∗∗*^*P* < 0.01, ^*∗∗∗*^*P* < 0.001.

It was reported that changes in the MC microenvironment of the mouse peritoneum could lead to the alteration of the release of proteases, membrane signaling, and membrane receptor phenotype in MC [12]. We next attempted to examine tryptase granules by immunohistochemistry. As shown in Figure 4(a), the relative level of tryptase granules in MC was significantly higher in the IBS group compared to that in the normal group (*p*=0.0028, Figure 4(a)). Compared with the IBS group, the level of tryptase particles was significantly reduced abdominal massage group (*p*=0.0016, Figure 4(a)). However, the application of the MC blocker did not show a further reducing effect (*p*=0.1269, Figure 4(a)).

Recent studies indicated that the overactivation of the PAR2-PKC pathway could mediate TRPV1 phosphorylation, which can modulate abnormal visceral pain perception and regulate visceral hypersensitivity in IBS [8]. We next detected the protein levels of PAR2 and PKC with western blot as well as the downstream signaling PIP2-PLC axis. Both PAR2 and PKC proteins were upregulated in the IBS group compared to the control group, and abdominal massage administration reduced their expression. Combined with abdominal massage, MC blockers could further suppress the expression levels of PAR2 and PKC (*p* < 0.001, Figure 4(b)). Similar results were observed for the PIP2-PLC axis, in which the proteins were upregulated in the IBS group and downregulated upon abdominal massage or MC blocker treatment (*p* < 0.001, Figure 4(c)). Together, these results suggest that abdominal massage may regulate the sensitivity of IBS by targeting the trypase-PAR2-PKC*ε* axis.

## 4. Discussion

In this study, using an IBS rat model, we demonstrated the beneficial effect of abdominal massage on alleviating IBS-induced histological damage. Abdominal massage tends to reduce the recruitment of mast cells into intestine tissues and preserve the integrity of mitochondria in cells of intestine tissues. We further showed that abdominal massage attenuated the activation of the trypase-PAR2-PKC*ε* axis induced by IBS.

Abdominal massage has been reported to promote the function of the gastrointestinal tract by enhancing digestion and absorption, strengthening physical fitness, and improving the immunity of individuals [13]. Previous studies showed that abdominal massage could significantly ameliorate the symptoms of constipation [14], insomnia [15], and fecal incontinence [16]. It was also reported that abdominal massage can serve as an alternative treatment for IBS [17]. However, the mechanisms of abdominal massage for IBS treatment remain unclear.

In our study, we found an elevated infiltration of mast cells in the intestinal mucosa of IBS rats, which could be suppressed by abdominal massage, and the application of an MC blocker enhanced the effect of abdominal massage. Since the infiltration of mast cells can lead to inflammatory damage of intestine tissue in IBS [18], these results suggest that abdominal massage could alleviate the inflammatory responses mediated by mast cells in IBS. Indeed, IBS induction caused damage to the mitochondria in the intestine tissues, which was largely attenuated by abdominal massage and MC blockers. These findings were consistent with a previous study from Tianjin University of Traditional Chinese Medicine, which showed regular abdominal massage reduces visceral hypersensitivity and preserves mitochondrial integrity by targeting GDNF and the PI3K/AKT signaling axis [19]. Furthermore, ketotifen, a systemic antihistamine for the treatment of allergic rhinitis and allergic bronchitis by antagonizing histamine H1 receptor [20], can promote the beneficial effect of abdominal massage in IBS. Therefore, these data suggest that the combination of abdominal massage and ketotifen can serve as a promising therapeutic strategy for IBS.

We also reported that TRPV1 was upregulated in IBS rats, which could be inhibited by abdomen massage. TRPV1 can serve as a signal transducer of noxious heat and capsaicin [21], which regulates the sensitivity of mast cells and visceral hypersensitivity [10]. Previous evidence also indicates that endogenous anti-inflammatory lipid mediators could suppress TRPV1 activation for IBS treatment [10]. These findings were consistent with our data, which suggest that TRPV1 upregulation may contribute to the onset of IBS, and abdominal massage attenuates the damage of IBS by suppressing TRPV expression.

Different from the study in the Rat model [19], we found that IBS induced the activation of the trypase-PAR2-PKC*ε* axis, which may underlie the sensitivity of IBS upon abdominal massage. It was reported that the overactivation of the PAR2-PKC*ε* pathway can promote the phosphorylation of TRPV1 protein and decrease the TRPV1 opening threshold, which can enhance abnormal visceral pain perception and ultimately lead to the occurrence of visceral hypersensitivity [21, 22]. Consistently, our data showed that the PAR2-PKC*ε* pathway was activated upon IBS induction. Our data further highlighted that ketotifen combined with abdominal massage can significantly suppress the activation of the PAR2-PKC*ε*-TRPV1 axis in the IBS rat model. However, it is worth mentioning that other signaling pathways may also regulate the sensitivity of IBS. For example, vitamin D supplementation has been shown to relieve the symptoms in IBS patients [23].

Our study suffers from the following limitations: (1) Abdominal massage in rats was performed by humans, which may cause psychological stress in rats and interfere with the interpretation of the data. (2) Our study only focused on the analysis of mast cells; however, the contribution of other immune cells such as macrophages to the inflammatory damages in IBS needs to be evaluated as well. (3) The physiological system of rats is different from that of humans; therefore, these findings need to be validated in human trials.

## 5. Conclusions

In summary, we found IBS induction in rat models leads to the infiltration of mast cells in intestine tissues, which were abrogated by an abdominal massage with or without ketotifen treatment. IBS induction upregulated TRPV1 and the trypase-PAR2-PKC*ε* axis, and abdominal massage could attenuate their activation in the IBS rat model. These findings suggest that abdominal massage produces a beneficial effect in alleviating IBS through reducing mast cell recruitment and attenuating the trypase-PAR2-PKC*ε* pathway.

## Data Availability

All the data generated and/or analyzed during this study are included in this published article. The data are available upon reasonable request.
